# Supplementary material for: New Smart Bioactive and Biomimetic Chitosan-Based Hydrogels for Wounds Care Management
Source: Pharmaceutics. 2023 Mar 17;15(3):975. doi: 10.3390/pharmaceutics15030975 (PMC10060009; doi:10.3390/pharmaceutics15030975)
Supplement: Supplementary file 1 [file pharmaceutics-15-00975-s001.zip › pharmaceutics-2219748-supplementary.pdf]

2.2. Methods

2.2.1. Synthesis of Oxidized Chitosan and Oxidized Hyaluronic Acid (oxCS, oxHA)

Chitosan (CS) and hyaluronic acid (HA) were oxidized using the periodate method, previously described in the literature [59,61]. Briefly, 1 g of polymers (CS, HA) were added to distilled water (1:100) and stirred at room temperature for 1 h. The sodium periodate solution ($NaIO_4$) 10% (w/w) was prepared ex tempore and 14 mL were added gradually into each polymer dispersion. Each mixture was stirred for 3 h, under magnetic stirring with 200 rpm, in a dark environment, at room temperature. Additional maintaining of 48 h for CS and 24 h for HA, respectively, in dark conditions, at room temperature, was necessary to complete the reactions. Finally, 3 mL of ethylene glycol was added to remove the unreacted $NaIO_4$ from each reaction. The purification of oxidized polymers was performed by dialysis against distilled water for five days, while the water was changed 3-4 times each day. Finally, oxCS and oxHA were lyophilized using a Christ freeze-dryer alpha 2–4 LSC at -50°C and 0.4 mbar. The obtaining process of oxidized polymers (oxCS, oxHA) is illustrated in Figure 1.

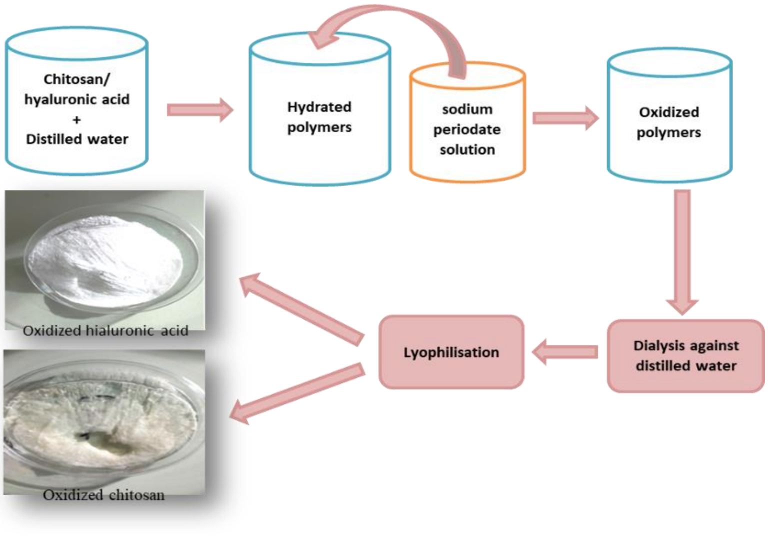

Figure S1. The obtaining process of oxCS and oxHA.

3.3. Physicochemical Characterization of Oxidized Polymers

FT-IR Spectroscopy

The native CS exhibits a large vibration band at 3550–3200 cm^{-1} as a result of overlapping stretching vibration of -OH and primary amine ($-NH_2$) groups. The peak around 2920 cm^{-1} results from the stretching vibration of C-H and around 1377 cm^{-1}, and the symmetric bending vibration of C-H was observed. The large band region within 1072-895 cm^{-1} is assigned to the D-glucosamine backbone. The stretching vibration of C=O and the bending vibration of N-H in acetyl groups are indicated by the presence of peaks around 1652-1555 cm^{-1} (Figure S2a) [73–75].

The spectra of oxCS show a new peak at 1732 cm^{-1} characteristic for the carbonyl group (C=O) stretch, compared with the spectra of native CS, which confirms that the oxidation reaction occurred properly. Also, the peak in the 2924-2854 cm^{-1} range which appears as a

shoulder-type peak just to the right of the alkyl C–H stretches, is specific for the H-C=O stretch. During the oxidation process several intra- and intermolecular interactions could occur between unreacted $-NH_2$ groups and new C=O groups. These interactions result in the formation of a dynamic Schiff base, iminols, and/or hemiacetals, which could overlap the oxidation signals. According to scientific data, the IR spectra of the Schiff bases exhibit medium to strong intensity absorption bands in the range 1615–1650 cm^{-1} as a consequence of C=N stretching vibrations [66,76].

For example, the new peak observed at 1620 cm^{-1} indicates the presence of an imino bond (C=N) and the new sharp peak from 3259 cm^{-1} in the O-H range, reconfirming the Schiff base presence and their possible tautomer iminol. The peak from 953-895 cm^{-1} could be assigned to hemiacetals interactions, which may be formed under periodate oxidation [70,77,78].

Referring to native HA, this polymer has three functional groups: carboxyl (-COOH), hydroxyl (primary and secondary O-H), and N-acetyl group (acetamide). The structure of HA is quite similar to CS, except for the absence of the free $-NH_2$ group. After the oxidation of HA, new clear peaks at 1732 cm^{-1} and at 2924-2854 cm^{-1} range appear, which confirm the presence of the carbonyl group, and so the success of the oxidation reaction [61]. Since HA does not contain any free $-NH_2$ groups, the intra- and intermolecular Schiff base interactions did not occur, in contrast to oxCS. However, the formation of hemiacetals is sustained due to the new peak observed at 876 cm^{-1} (Figure S2b)

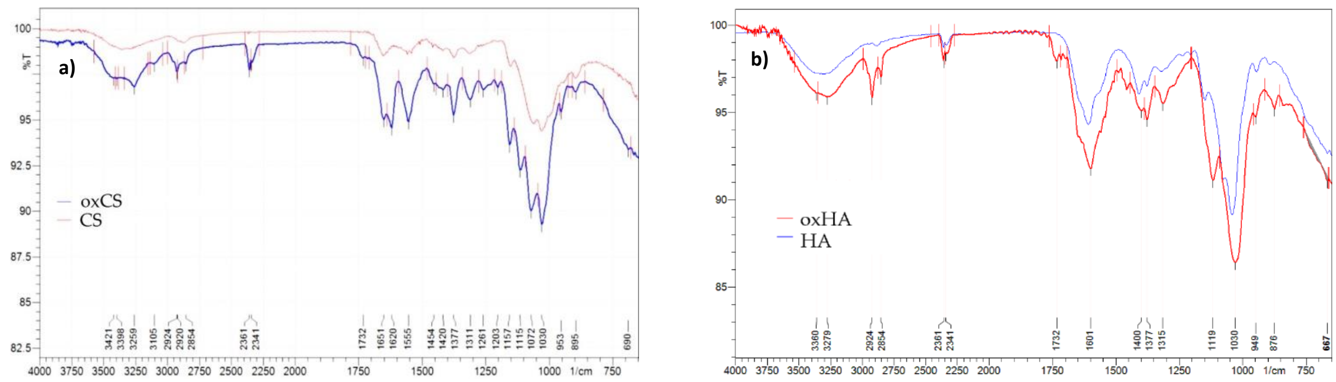

Figure S2. (a) FT-IR spectra of native (CS, HA) and (b) oxidized (oxCS, oxHA) polymers.

NMR Spectroscopy

In the ^{1}H-NMR spectra of oxCS (Figure S3b) the lack of the signal from 3.094 ppm and the presence of a signal at 8.157 ppm, in reference with the ^{1}H-NMR spectra of native CS (Figure S3a), demonstrate that the oxidation took place [76,79]. In addition, the carbon signal at 165.64 ppm from the ^{13}C-NMR spectra of oxCS (Figure 6d), characteristic of the aldehyde group, strongly supports the structure of oxCS [80].

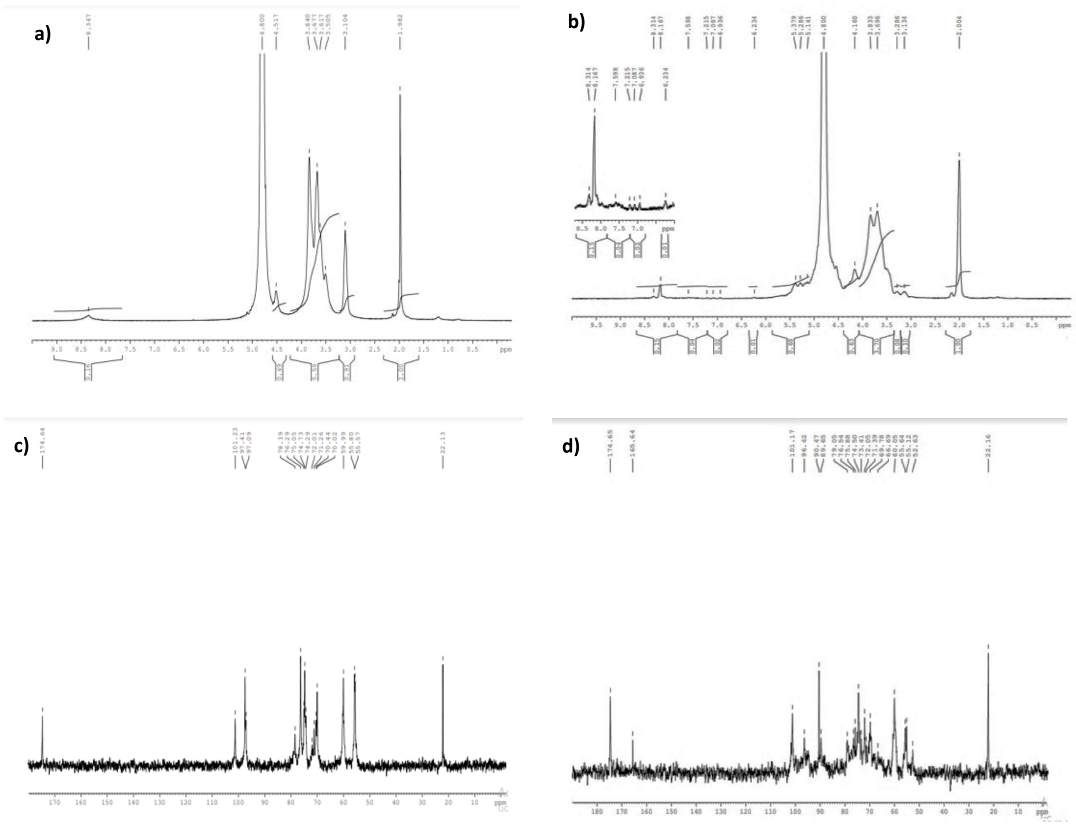

Figure S3. ^{1}H-NMR and ^{13}C-NMR spectra of CS (a, c) and oxCS (b, d).

For oxHA in the ^{1}H-NMR spectra (Figure S4b), the presence of aldehyde protons was confirmed by the appearance of chemical shifts between 8 and 9.5 ppm compared to the HA spectra (Figure S4a) [61,80]. Moreover, the carbon of the aldehyde group appears as a new signal at 173 ppm (Figure S4d), which indicates both sp^{2} hybridization and the double bond between carbon and oxygen [32,33].

The oxidation degree for oxHA was 8.5%, quantified by comparing the integration ratio for the signal protons of the $-CH_3$ group and the aldehyde proton (C-H) [81]. In the case of oxCS, due to the intra- and intermolecular interactions described previously, the determination of oxidation degree through NMR spectroscopy was not properly quantified.

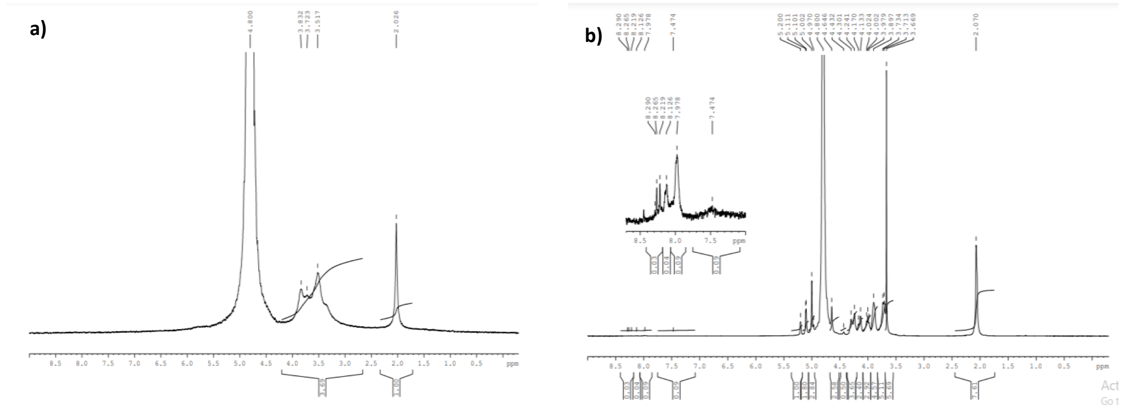

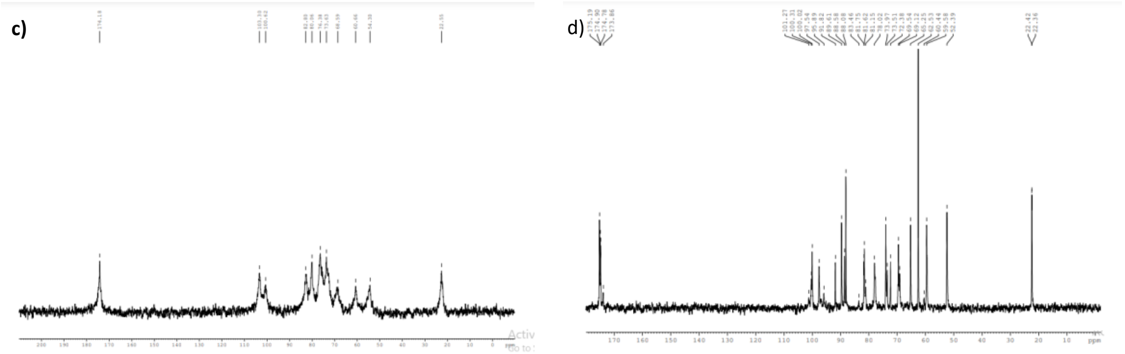

Figure S4. ^{1}H-NMR and ^{13}C-NMR spectra of HA (a, c) and oxHA (b, d).
